# Differential epithelial expression of the putative innate immune molecule SPLUNC1 in Cystic Fibrosis

**DOI:** 10.1186/1465-9921-8-79

**Published:** 2007-11-07

**Authors:** Lynne Bingle, Frances A Barnes, Simon S Cross, Doris Rassl, William A Wallace, Michael A Campos, Colin D Bingle

**Affiliations:** 1 Department of Oral Pathology, School of Clinical Dentistry, University of Sheffield, Sheffield, UK; 2 Academic Unit of Respiratory Medicine, University of Sheffield Medical School, Sheffield, UK; 3 Academic Unit of Pathology, University of Sheffield Medical School, Sheffield, UK; 4 Department of Pathology, Papworth Hospital, Cambridge, UK; 5 Department of Pathology, University of Edinburgh, Edinburgh, UK; 6 Division of Pulmonary and Critical Care Medicine, University of Miami, Miami, Florida, USA

## Abstract

**Introduction:**

Short PLUNC1 (SPLUNC1) is the founding member of a family of proteins (PLUNCS) expressed in the upper respiratory tract and oral cavity, which may function in host defence. It is one of the most highly expressed genes in the upper airways and the protein has been detected in sputum and nasal secretions. The biology of the PLUNC family is poorly understood but in keeping with the putative function of the protein as an immune defence protein, a number of RNA and protein studies have indicated that SPLUNC1 is increased in inflammatory/infectious conditions such as Cystic Fibrosis (CF), COPD and allergic rhinitis.

**Methods:**

We used immunohistochemistry to localise SPLUNC1 in lung tissue from patients with CF and a range of other lung diseases. We used a range of additional markers for distinct cell types to try to establish the exact site of secretion of SPLUNC1. We have complemented these studies with a molecular analysis of SPLUNC1 gene expression in primary human lung cell cultures and isolated inflammatory cell populations.

**Results:**

In CF, expression of SPLUNC1 is significantly elevated in diseased airways and positive staining was noted in some of the inflammatory infiltrates. The epithelium of small airways of CF lung exhibit significantly increased SPLUNC1 staining compared to similar sized airways in non-CF lungs where staining is absent. Strong staining was also seen in mucous plugs in the airways, these included many inflammatory cells. No alveolar epithelial staining was noted in CF tissue. Airway epithelial staining did not co-localise with MUC5AC suggesting that the protein was not produced by goblet cells. Using serial sections stained with neutrophil elastase and CD68 we could not demonstrate co-localisation of SPLUNC1 with either neutrophils or macrophages/monocytes, indicating that these cells were not a source of SPLUNC1 in the airways of CF lungs. No change in staining pattern was noted in the small airways or lung parenchyma of other lung diseases studied including, COPD, emphysema or pneumonia where significant NE and CD68 staining was noted. Cultures of primary tracheobronchial epithelial cells were analysed by RT-PCR and showed that pro-inflammatory mediators did not induce expression of SPLUNC1. We have also shown that SPLUNC1 gene expression was not seen in isolated human mononuclear cells, macrophages or neutrophils.

**Conclusion:**

These studies show that SPLUNC1 is specifically and significantly increased in the small airways of lungs from patients with CF. They further suggest that it is the airway epithelium that is responsible for the increased levels of SPLUNC1 in CF and not inflammatory cells; this could be a defensive response to the infectious component of the disease.

## Background

SPLUNC1 is the founder member of the PLUNC family of putative innate immune molecules, and is highly expressed in the epithelium of the upper respiratory tract, nasopharynx and submucosal glands [1]. The mouse orthologue of SPLUNC1, Palate Lung Nasal Clone (plunc) was first identified as a molecule expressed in the developing mouse oral cavity around the time of palatal shelf closure; it is expressed in the nasal epithelium of the mouse embryo and the trachea and bronchi of adult mouse lung [2]. Using a systematic, bioinformatic and expression study we subsequently identified the wider PLUNC family of nine human genes located on chromosome 20 [3,4]. PLUNC genes are expressed in overlapping patterns predominantly in the upper respiratory tract, nasal passages and oral cavity [1,3]. All PLUNC proteins contain signal peptides suggesting they would be secreted into the extracellular fluids bathing these locations. Due to the structural similarity between PLUNCs and the lipid/LPS binding, innate defence proteins lipopolysaccharide binding protein (LBP) and Bactericidal/permeability-increasing protein (BPI), we, and others, have hypothesised that PLUNCs may function in the innate immune defence of the respiratory tract. [3-7]. However, direct proof of such a host defence function remains to be published. SPLUNC1 mRNA has been identified in tracheal epithelium [8,9] and in sub-mucosal glands and ducts [9]. The protein was localised to the same sites [10], and our previous studies have shown that the submucosal glands of the upper respiratory tract and the minor mucosal glands of the oropharynx appear to be the major sites of protein localisation [11]. These same studies also show that expression of SPLUNC1 is limited to a few non-ciliated epithelial cells of the upper airways and is absent from small airways and from peripheral lung [11]. This contrasts to the situation in the mouse where in situ hybridisation suggests that *splunc1 *is expressed in the majority of epithelial cells in the trachea and main bronchi [1,8]. SPLUNC1 is one of the major proteins secreted from differentiated human tracheo-bronchial epithelial cells in culture [10] and has been identified as a highly expressed gene in such cells in a number of studies [12,13].

Cystic fibrosis (CF) is an autosomal recessive disorder caused by mutations of the CF trans-membrane regulator (CFTR) gene [14,15]. The genetic defect in CF leads to abnormal epithelial chloride and water transport and this results in increased viscosity of, and subsequent decreased clearance of, airway secretions. This in turn affects airway defence resulting in chronic lung infection [16]. Inflammatory cells recruited in response to this active infection lead to a state of chronic airway inflammation. CF lung disease is therefore characterised by plugging of airways associated with persistent bacterial infection and massive neutrophil infiltration [16]. The CF lung becomes infected with a distinctive bacterial flora, including *Pseudomonas aeruginosa*, *Staphylococcus aureus*, and organisms of the *Burkholderia cepacia *complex which are associated with plugging of the small airways. This plugging directly contributes to the impaired lung function seen in CF, leading to respiratory failure. CF lung disease is largely restricted to the airway compartment of the lung with the parenchyma being largely unaffected. There is no effective treatment for CF and patients with the disease will ultimately require lung transplantation [16]. Innate immune defences of the lung are significantly impaired in CF as a consequence of a combination of factors, including phenotypic alterations of the airway epithelium, the elevated viscosity, alterations in the ionic strength and pH of the airway lining fluid and the increased levels of inflammatory cells present in the inflamed tissues [16]. The defects in innate defences become self-sustaining, because the inflammatory cell derived proteases, including neutrophil elastase (NE), released from the abundant mass of recruited cells, specifically degrade many host defence proteins [17].

As a putative innate defence molecule expressed in the upper airways and in submucosal glands, SPLUNC1 could potentially be involved in combating the chronic infections seen in CF and other lung diseases with an infectious component. Previous studies have shown that SPLUNC1 is elevated in the sputum of patients with COPD [9]. Although similar studies have not been shown in CF, a proteomic study has shown that CF nasal epithelial cells contain increased levels of SPLUNC1 [18] and molecular studies have shown that CF epithelial cells appear to express abundant SPLUNC1 [12]. In this paper we demonstrate that CF airways specifically express abundant SPLUNC1 and show that this elevated protein expression is not the product of the inflammatory cells that accumulate in the disease.

## Methods

### Immunohistochemistry

The tissue used in this study was collected with ethical approval only on a fully anonymised basis and thus we have no further patient details. Serial sections were cut from formalin-fixed and paraffin-embedded tissue as described [11]. For normal tissues, sections were taken so as to be as representative of normal architecture as possible, although as all tissues were removed for medical reasons there was some evidence of disease in some sections. Sections from the major bronchi and peripheral lung were cut from 10 cases of normal lung and similar samples were also obtained from 10 patients with CF who were undergoing lung transplantation. We also studied 10 cases from patients with emphysema and two cases from patients with pneumonia. The histologically "normal" and emphysematous samples were resections taken during surgery for cancer, where no tumour tissue was seen to be present, the CF tissue was removed during the course of transplantation and the pneumonia tissue was collected during post-mortem. The slides were treated with 2% hydrogen peroxide in methanol for 20 minutes to quench endogenous peroxidase.

The following antibodies were used in this study: A polyclonal antibody raised against human SPLUNC1 [10] (final dilution 1:300); a polyclonal antibody to human mucin 5AC (MUC5AC, a gift from David Thornton, University of Manchester, UK; final dilution 1:250); monoclonal antibodies to tryptase, CD68 and neutrophil elastase purchased from Dako (1:1200, 1:400 and 1:300 respectively). A standard antigen retrieval procedure using tri-sodium citrate in a microwave for 8 minutes was used for the MUC5AC, tryptase and CD68 antibodies. The specificity of the SPLUNC1 antibody has previously been shown by western blotting using a number of recombinant PLUNC proteins [11]. Sections were incubated with 100% normal serum (goat for polyclonal antibodies, horse for monoclonal antibodies) at room temperature for 30 minutes and then at 4°C overnight with the antibodies diluted as indicated above with 100% normal serum. Rabbit or mouse IgG (DAKO) was used as a negative controls on replicate slides. A Vectastain Elite ABC kit (Vector Laboratories) containing an appropriate biotin-labelled secondary antibody was used according to the manufacturer's instructions. Peroxidase enzymatic development was performed using, a Vector NovaRed substrate kit resulting in red staining in positive cells. Sections were counterstained with haematoxylin, dehydrated to xylene and mounted in DPX.

### Tissue culture, Cell isolation, RNA extraction and RT-PCR

Human tracheobronchial epithelial (TBE) cell cultures were prepared by methods described previously [19] using trachea and bronchi from lungs that were not deemed suitable for transplant through the Life Alliance Organ Recovery Agency of the University of Miami and approved by the local institutional review board. Cells were grown in a medium containing 50% DMEM and 50% LHC basal medium (Biosource International, Camarillo, CA, USA) supplemented with hormones and trace elements as described [19]. Upon reaching confluence (after 3–7 d), the medium from the apical surface was removed, leaving the top surface exposed to air (air-liquid interface cultures, ALI). To study the effect of retinoic acid (RA) on the expression of SPLUNC1, normal RA (50 nM) was removed from the medium of fully differentiated TBE cells after 14 days exposed to air. To study the effects of pro-inflammatory mediators on SPLUNC1 gene expression, TBE cells were stimulated with 25 ng/ml of IL-1β or TNFα (both from R & D Systems, Inc, Minneapolis, MN, USA) for 6, 24 or 48 hours prior to harvest. Stimulations for RNA analysis were performed on cultures from three individual donors.

Human peripheral blood neutrophils, mononuclear (MNC) cells, monocytes and T and B cell enriched cell populations from normal healthy donors, were isolated by density gradient centrifugation and cultured as previously described [20]. Monocyte-derived macrophages were differentiated from monocytes on tissue culture plastic using standard protocols [21]. A SPLUNC1 expressing CHO cell line was generated from a full-length human SPLUNC1 expression clone [11] using the Flp-in system (Invitrogen).

Total RNA was isolated as previously described [1]. Reverse transcription was also performed as previously described [22] in a total volume of 25 μl using an oligo-dT primer and 1 μg of total RNA. PCR reactions were performed using 1 μl of each reaction product and the following primer pairs. SPLUNC1F 5' ATG CCC TCA GCA ATG GCC TGC T 3', SPLUNC1R 5' GTG AGG CTG TCC AGA AGA CC 3': WFDC2 F: 5' CGG CTT CAC CCT AGT CTC AG 3'; WFDC2 R: 5' AAA GGG AGA AGC TGT GGT CA 3'; Elafin F: 5' ACC TTC CTG ACA CCA TGA GG 3'; Elafin R: 5' GAT GAG AGA GGC AGC TCC AG 3'; BCL2A1 F 5' GCC ACC ATG ACA GAC TGT GAA TTT GGA TAT 3', BCL2A1R 5' TCA ACA GTA TTG CTT CAG GAG AGA 3': Primer pairs were designed using Primer3 [23] and were designed to cross intro/exon boundaries. 30 cycles of the following program (94°C for 1', 60°C for 2' and 72°C for 3') generated the appropriately sized products, which were resolved on 2% TAE agarose gels, stained with ethidium bromide and photographed. Representative samples of each were directly cloned in TOPO pCRII (Invitrogen) and sequenced.

## Results

**Figure 1:**
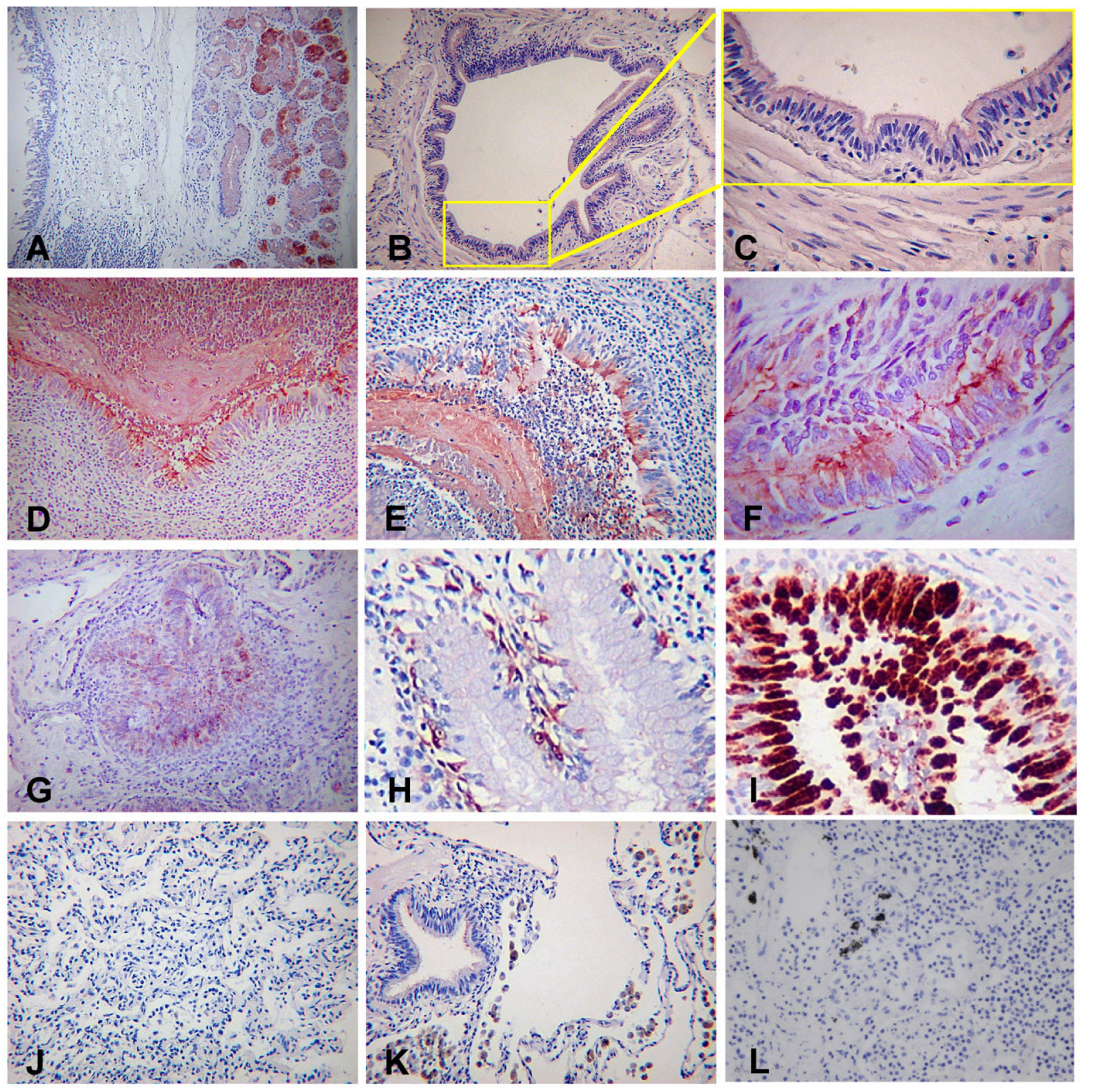
**SPLUNC1 is increased in the small airways of CF lung**. Immunohistochemical localisation of SPLUNC1 was performed as described in the materials and methods sections. Sections show submucosal gland staining in "normal" human airways (A) and a lack of staining in the epithelium of smaller airways (B, C, an enlarged inset of B). Expression of SPLUNC1 is increased in cases of CF (D-H) where staining was predominantly found in the epithelial cells of the smaller airways and also within the inflammatory cell containing mass within the plugged lumens. SPLUNC1 did not co-localise with MUC5AC (I). There was no staining within the peripheral lung tissue of cases of CF (J). Cases of emphysema (K) and bacterial pneumonia (L) also did not exhibit SPLUNC1 staining.

On the basis of a number of reports which have shown that SPLUNC1 may be elevated in inflammatory lung disease, we used immunohistochemistry to study SPLUNC1 protein expression in chronically inflamed lung tissue from patients with a range of conditions including those who had undergone transplantation for CF. We have previously shown that SPLUNC1 is predominantly localised in the minor mucosal glands of the oro- and naso pharynx, whilst in the airways SPLUNC1 is found in a few epithelial cells of the large airways but is most strongly expressed in the submucosal glands [11] (Figure 1A). In histologically "normal" tissue from patients undergoing lobectomy surgery, SPLUNC1 staining is not seen in smaller airways (Figure 1B and 1C). In large airways of subjects with CF, the site or level of staining of SPLUNC1 within the bronchiolar epithelial cells or the submucosal glands was similar to that of normal lungs (results not shown). However, the situation in smaller airways within the peripheral lung of the CF cases was markedly different (Figure 1D, E, F, G, H). In this study we define small airways as those without cartilage or submucosal glands. In all 10 cases there was significant staining in the abnormal (hyperplastic) epithelium. This staining appeared to be restricted to the epithelium (Figure 1D, E, F, G, H) and contrasts greatly to the situation seen in similar sized airways from CF-disease free lung (Figure 1B). Epithelial staining appeared to be present towards the apical surface of the cells and along the epithelial surface. In addition to the greatly increased staining seen within the epithelium, the inflammatory plugs within the airway lumen were also found to stain strongly for SPLUNC1 (Figure 1D, E, G) suggesting the protein is secreted into the lumen of these diseased airways. Although significant numbers of inflammatory cells were present in these sections it was not immediately clear if these cells were expressing SPLUNC1. SPLUNC1 was not present in the increased mucous cell population found in some regions of the diseased airways (Figure 1H). This observation was confirmed when serial sections were stained with the goblet cell marker MUC5AC (Figure 1I). These results clearly show that SPLUNC1 immunoreactivity is increased in the small airways of the lungs of patients with CF and furthermore suggest the protein is secreted into the luminal contents of the diseased lung. The specificity of this was highlighted by the observation that peripheral lung sections from the same CF patients were uniformly negative (Figure 1J), as were additional sections from patients with emphysema (Figure 1K) and bacterial pneumonia (Figure 1L). In all of these conditions a significant number of inflammatory cells (predominantly neutrophils and monocytes/macrophages) were found and these did not appear to stain with SPLUNC1.

**Figure 2:**
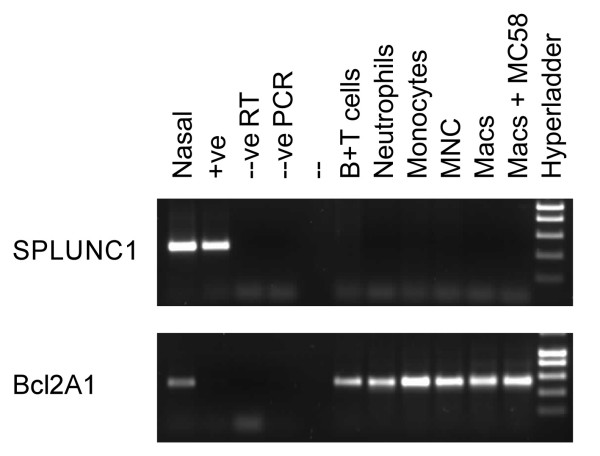
***SPLUNC1 *is not expressed in neutrophils, monocytes or macrophages**. Expression of *SPLUNC1 *was investigated by the use of RT-PCR with exon spanning primer pairs as described in the materials and methods section. Total RNA from B and T cells, neutrophils, monocytes, mononuclear cells and macrophages (either mock treated or infected with *Neisseria meningitidis *mc58) as well as positive control tissues (nasal epithelium and SPLUNC1-CHO cells), were used as template. The negative controls were a negative reverse transcription reaction performed in the absence of RT enzyme and a PCR reaction performed without Taq polymerase. Primers to the myeloid enriched anti apoptotic gene Bcl2A1 were used as a positive control for all samples.

**Figure 3:**
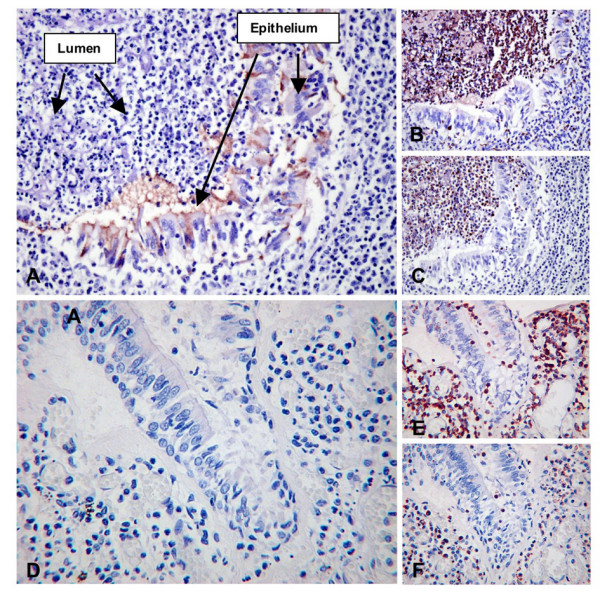
**SPLUNC1 is not expressed in inflammatory cells in CF or pneumonia**. Serial sections of lung tissue were stained as described in the materials and methods section. SPLUNC1 (A) is not found to be expressed in CD68 expressing cells (Macrophages, B) nor in neutrophil elastase expressing cells (Neutrophils, C) in CF. Arrows in A point to the SPLUNC1 negative lumen as well as the positively staining epithelial cell layer. In sections from a case of bacterial pneumonia neither macrophages (E) nor neutrophils (F) appear to stain for SPLUNC1 (D), which is negative in this field.

As BPI, one of the paralogues of SPLUNC1, is highly expressed in neutrophils [24] and because it has recently been suggested that SPLUNC1 may be present in peripheral blood neutrophils [25] we examined this expression in more detail. Initially we took isolated populations of inflammatory cells and used them for a series of RT-PCR experiments. RNA isolated from peripheral blood neutrophils, mononuclear cells, B and T cells, monocytes and monocyte derived macrophages (MDMs, both mock infected and infected with Neisseria meningitidis MC58) were all negative for SPLUNC1 expression (Figure 2, upper panel). A strong positive signal was seen in samples from nasal septal epithelium and a stable CHO cell line expressing human SPLUNC1. All of the inflammatory cell samples were shown to be positive for the apoptosis regulator Bcl2A1 (Figure 2, lower panel); this is expressed in leukocytes [26]. Our results suggest that neutrophils and other leukocytes do not express SPLUNC1 RNA. To exclude the possibility that SPLUNC1 RNA is present only in immature neutrophils as they undergo differentiation in the bone marrow and prior to release into the circulation we examined the data set of Theilgaard-Monch et al [27] in which the differentiation programme of neutrophils was examined by expression array. In these studies no SPLUNC1 transcripts were detected at any stage of differentiation in three individual experiments using different donors (results not shown). This observation was subsequently confirmed by the failure to detect SPLUNC1 mRNA in similar samples by northern blotting (Jack Cowland, personal communication). To further confirm the lack of SPLUNC1 in inflammatory cells within in the CF lung we stained serials tissue sections with SPLUNC1, neutrophil elastase (as a marker for neutrophils) and CD68 (as a marker for macrophages/monocytes) (Figure 3). In these studies it can clearly be seen that the staining for SPLUNC1 is either within the occluded lumen or within the epithelial cells (Figure 3A) whereas CD68 (Figure 3B) and neutrophil elastase (Figure 3C) are predominantly seen within the inflammatory cell mass in the airway lumen or in isolated cells either infiltrating the epithelium or within the sub epithelial layer. The lack of SPLUNC1 staining in sections of tissue from bronchial pneumonia (Figure 3D) contrasts strikingly with the intense staining of both macrophages and neutrophils (Figure 3E, F). These results suggest that the increased production of SPLUNC1 in CF small airways was unlikely to have arisen from inflammatory cells.

**Figure 4:**
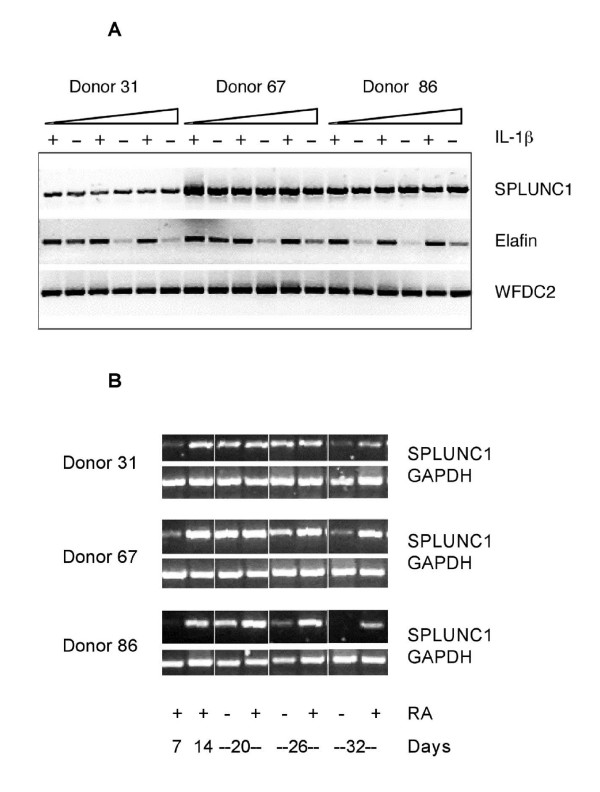
**Expression of *SPLUNC1 *in TBE cells grown at the ALI is not induced by pro-inflammatory mediators but requires continued RA driven differentiation**. **A**. TBE cells isolated from three different donors were grown at the ALI as described in the materials and methods section. Cells were treated for increasing lengths of time with 25 ng/ml of IL-1β prior to isolation of RNA. Expression of *SPLUNC1 *was investigated by the use of RT-PCR with exon spanning primer pairs as described in the materials and methods section. Primers to Elafin and WFDC2 were used as controls. **B**. The effect of withdrawal of RA from the ALI growth medium was tested in TBE cells from the same three donors. ALI cells were established using standard grow conditions in medium containing 50 nM all trans retinoic acid (RA). After 14 days in culture RA was removed from the medium of one group of cells and culture was continued for an additional 18 days. RNA was harvested at 7 and 14 days during ALI growth and 20, 26 and 32 days following RA removal for 6, 12 and 18 days respectively prior to the isolation of RNA. Expression of *SPLUNC1 *was investigated by the use of RT-PCR with exon spanning primer pairs as described in the materials and methods section with primers to GAPDH serving as a positive control.

As the CF lung is a hyper-inflammatory environment, we studied SPLUNC1 gene expression in tracheobronchial epithelial cells, as a surrogate for the situation seen *in vivo*, to determine whether it was influenced by classical pro-inflammatory cytokines. SPLUNC1 is highly expressed in tracheobronchial epithelial cells when they are differentiated at an air liquid interface [9,10,13]. We studied the potential of these cells to increase SPLUNC1 mRNA following treatment with IL-1β for 6, 24 and 48 hours. Although there is a level of variability of SPLUNC1 expression between cells isolated from different donors, levels of SPLUNC1 mRNA were not elevated by IL-1β treatment at any of the time points studied (Figure 4A). We could show, however, that these stimulations did induce expression of the gene for the antiproteinase, Elafin and this is consistent with our previous studies [28]. Also consistent with our previous results the related host defence molecule WFDC2 was not induced in these cells. TNFα treatment of the same cultures failed to induce expression of SPLUNC1 (results not shown). We were also unable to show any induction of SPLUNC1 mRNA in similar cell culture experiments following exposure to either bacterial lipopolysaccharide (LPS) or human neutrophil elastase, a neutrophil derived protein that is abundant in the CF lung (results not shown). These results suggest that the increase in SPLUNC1 staining in the epithelium of the CF airway is not due to a transcriptional effect of cytokines or pro-inflammatory mediators acting directly on the SPLUNC1 gene. It may, however, be due to phenotypic alterations of the epithelial cell populations in the airways leading to a greater number of SPLUNC1 positive cells being present. In support of this suggestion we have shown that TBE cells cultured at the ALI require a source of RA in the culture media to maintain continued levels of SPLUNC1 expression (Figure 4B). Removal of RA from the basal media of differentiated cell cultures leads to a progressive loss of SPLUNC1 over an 18-day period. We have previously shown that the withdrawal of RA leads to an increase in expression of Elafin and a loss of MUC5AC [28]; this is known to correspond with a return to a squamous (de-differentiated) cell phenotype.

## Discussion

Since our original description of the expression pattern of human SPLUNC1 gene being limited to the upper respiratory tract [1] a number of other expression studies have shown that it is highly expressed in the tracheobronchial epithelium of the human respiratory tract. A number of proteomic and protein studies have shown that the protein can be detected in nasal secretions [29-32] where levels may be altered by chemical injury [29,30] and viral infection [33]; it has also been studied in sputum, where levels appear to be increased in COPD [9]. A large-scale EST sequencing and characterisation study appeared to show that the SPLUNC1 gene was more highly expressed in airway epithelial cells from CF patients compared to non-CF patients [12]. Additionally, CF nasal epithelial cells have also been shown to contain increased levels of SPLUNC1 compared to normals [19].

More recently we performed an extensive study of the localisation of SPLUNC1 protein in normal human tissues from the respiratory tract, oral cavity and nasopharynx [11]. These studies confirmed previous findings that the gene was expressed in submucosal glands and some cells of the tracheal epithelium but extended our observations to show that the protein was expressed in multiple minor mucosal glands from the nasal antrum and throughout the oral cavity including glands localised in the tongue and adjacent to tonsils. This study also showed that small airways, that is those which contain neither cartilage nor submucosal glands and peripheral lung tissue, do not express SPLUNC1 [11]. In striking contrast to the situation described for normal lung, our present study has clearly shown that SPLUNC1 is significantly increased in the small airways of patients with advanced CF. The specificity of this observation is shown by the fact that similar alterations are not seen in patients with other lung conditions, including bacterial pneumonia and emphysema, as well as tuberculosis (results not shown). It is also noteworthy that this change in abundance of SPLUNC1 in CF is found at sites where the protein is not normally seen. Our studies suggest that this is likely due to an increase in epithelial production of the protein as we have been unable to show that SPLUNC1 is present in the inflammatory cells that accumulate within the diseased airways in this condition. Indeed no SPLUNC1 staining is seen in the peripheral lung tissue of the same CF patients nor in the peripheral lung of patients with bacterial pneumonia, both of which contain significant numbers of neutrophils, monocytes and macrophages. We were also unable to show that SPLUNC1 mRNA was present in isolated human neutrophils, monocytes or monocyte-derived macrophages. As the transcription of many neutrophil gene products is largely restricted to the period in which the cells are developing within the bone marrow [27,33] we also looked at expression in highly purified cell populations from this source. Again, these samples were negative for SPLUNC1 mRNA. On the basis of these observations we suggest that SPLUNC1 is not a significant product of inflammatory cells in the diseased airway.

We have been also been unable to show that treatment of airway cells with the classical inflammatory mediators, IL-1β and TNFα (nor indeed bacterial LPS or hNE) affects SPLUNC1 gene expression suggesting that the gene is not transcriptionally regulated by these factors. This is consistent with a recent report showing a similar lack of induction of the SPLUNC1 in nasal epithelial cell cultures treated with the same stimuli [34]. On the basis of these results we would suggest that the increased SPLUNC1 that is seen in the epithelial cells of the CF airways is the result of a phenotypic alteration in the epithelium itself. It is well known that airway epithelial phenotypic changes are associated with changes in gene expression and this has been well studied in tissue culture models. Our data show that SPLUNC1 gene expression in airway epithelium in culture requires a continued exposure to RA as this maintains a mucociliary phenotype. This observation is consistent with other published data [9,10], including the study of Ross et al, that shows that SPLUNC1 is one of the most differentially expressed genes during this differentiation process [13]. In our cultures we have shown that SPLUNC1 is present in non-ciliated cells (results not shown) but *in vivo *it is clear that the protein is only expressed in a limited number of epithelial cells [11] and is not located in goblet cells.

Two significant questions arise from our observations. Firstly, what is the consequence of this alteration in SPLUNC1 production in CF and secondly, are levels of SPLUNC1 increased in less severe CF lung disease? In line with the hypothesised function of SPLUNC1 as an innate immune defence protein, it might be expected that the protein would be increased as a defensive response in severe CF where the pathogenic load is markedly elevated. It is clear that other innate defence factors are also increased in this condition but perhaps the severity of the inflammatory disease coupled with the significant pathogenic load overcomes the defensive capability of the innate immune shield. One of the major limitations of this study is that we have only been able to obtain tissue from patients with severe CF at the time of lung transplantation. It remains to be seen if similar alterations in SPLUNC1 localisation and expression are found in less severe cases; this could be accomplished in future studies by examining levels of SPLUNC1 protein in CF secretions.

## Conclusion

These studies show that the putative innate immune molecule, SPLUNC1 is specifically and significantly increased in the small airways of lungs from patients with CF. They further suggest that it is the airway epithelium that is responsible for the increased levels of SPLUNC1 in CF and not inflammatory cells; this could be a defensive response to the infectious component of the disease.

## Abbreviations

BPI Bactericidal Permeability Increasing Protein

CF Cystic Fibrosis

CHO Chinese Hamster Ovary

COPD Chronic Obstructive Pulmonary Disease

IL-1β Interleukin-1β

LBP Lipopolysaccharide Binding Protein

LPS Lipopolysaccharide

MDM Monocyte-derived Macrophages

MNC Mononuclear Cells

MUC5AC Mucin 5AC

NE Neutrophil Elastase

PLUNC Palate Lung Nasal Clone

RA Retinoic Acid

SPLUNC1 Short PLUNC 1

TBE Tracheobronchial epithelium

TNFα Tumour Necrosis Factor α

## Competing interests

The author(s) declare that they have no competing interests.

## Authors' contributions

**LB**: participated in the design and coordination of the study, carried out all of the immunohistochemical studies, performed gene expression studies and co-authored the draft of the manuscript.

**FAB**: carried out RT-PCR studies and contributed to the manuscript

**SSC**: provided invaluable histology expertise, took the photomicrograph and contributed to the manuscript

**DR**: provided the cystic fibrosis tissues, analysed the immunohistochemistry of these tissues and contributed to the manuscript.

**WAW**: provided the normal lung tissues, analysed the immunohistochemistry of these tissues, and contributed to the manuscript.

**MAC**: facilitated the culture of the TBE cells, provided the SPLUNC1 antibody and contributed to the manuscript.

**CDB**: conceived of the study, participated in the design and coordination of the study, performed gene expression studies and co-authored the draft of the manuscript.

All authors read and approved the final manuscript.

## References

[B1] Bingle CD, Bingle L (2000). Characterisation of the human plunc gene, a gene product with an upper airways and nasopharyngeal restricted expression pattern. Biochim Biophys Acta.

[B2] Weston WM, LeClair EE, Trzyna W, McHugh KM, Nugent P, Lafferty CM, Ma L, Tuan RS, Greene RM (1999). Differential display identification of plunc, a novel gene expressed in embryonic palate, nasal epithelium, and adult lung. J Biol Chem.

[B3] Bingle CD, Craven CJ (2002). PLUNC: a novel family of candidate host defence proteins expressed in the upper airways and nasopharynx. Hum Mol Genet.

[B4] Bingle CD, LeClair EE, Havard S, Bingle L, Gillingham P, Craven CJ (2004). Phylogenetic and evolutionary analysis of the PLUNC gene family. Protein Science.

[B5] Sung YK, Moon C, Yoo JY, Moon C, Pearse D, Pevsner J, Ronnett GV (2002). Plunc, a member of the secretory gland protein family, is up-regulated in nasal respiratory epithelium after olfactory bulbectomy. J Biol Chem.

[B6] Wheeler TT, Haigh BJ, McCracken JY, Wilkins RJ, Morris CA, Grigor MR (2002). The BSP30 salivary proteins from cattle, LUNX/PLUNC and von Ebner's minor salivary gland protein are members of the PSP/LBP superfamily of proteins. Biochim Biophys Acta.

[B7] Bingle CD, Craven CJ (2004). Meet the relatives: a family of BPI and LBP-related proteins. Trends in Immunology.

[B8] LeClair EE, Nguyen L, Bingle L, MacGowan A, Singleton V, Ward SJ, Bingle CD (2001). Genomic organization of the mouse plunc gene and expression in the developing airways and thymus. Biochem Biophys Res Commun.

[B9] Di Y-P, Harper R, Zhao Y, Pahlavan N, Finkbeiner W, Wu R (2003). Molecular cloning and characterization of spurt, a human novel gene that is retinoic acid-inducible and encodes a secretory protein specific in upper respiratory tracts. J Biol Chem.

[B10] Campos MA, Abreu AR, Nlend MC, Cobas MA, Conner GE, Whitney PL (2004). Purification and characterization of PLUNC from human tracheobronchial secretions. Am J Resp Cell Mol Biol.

[B11] Bingle L, Cross SS, High AS, Wallace WA, Devine DA, Havard S, Campos MA, Bingle CD (2005). SPLUNC1 (PLUNC) is expressed in glandular tissues of the respiratory tract and in cancers with a glandular phenotype. J Path.

[B12] Scheetz TE, Zabner J, Welsh MJ, Coco J, Eyestone M, Bonaldo M, Kucaba T, Casavant TL, Soares MB, McCray PB (2004). Large scale gene discovery in human airway epithelia reveals novel transcripts. Physiol Gen.

[B13] Ross AJ, Dailey LA, Brighton LE, Devlin RB (2007). Transcriptional Profiling of Mucociliary Differentiation in Human Airway Epithelial Cells. Am J Respir Cell Mol Biol.

[B14] Rommens JM, Iannuzzi MC, Kerem B (1989). Identification of the cystic fibrosis gene: chromosome walking and jumping. Science.

[B15] Riordan JR, Rommens JM, Kerem B (1989). Identification of the cystic fibrosis gene: cloning and characterization of complementary DNA. Science.

[B16] Rowe SM, Miller S, Sorscher EJ (2005). Cystic fibrosis. N Engl J Med.

[B17] Carroll TP, Greene CM, Taggart CC, McElvaney NG, O'Neill SJ (2005). Interleukin-1, Neutrophil Elastase, and Lipopolysaccharide: Key Proinflammatory Stimuli Regulating Inflammation in Cystic Fibrosis. Current Respiratory Medicine Review.

[B18] Roxo-Rosa M, da Costa G, Luider TM, Scholte BJ, Coelho AV, Amaral MD, Penque D (2006). Proteomic analysis of nasal cells from cystic fibrosis patients and non-cystic fibrosis control individuals: search for novel biomarkers of cystic fibrosis lung disease. Proteomics.

[B19] Nlend MC, Bookman RJ, Conner GE, Salathe M (2002). Regulator of G-protein signaling protein 2 modulates purinergic calcium and ciliary beat frequency responses in airway epithelia. Am J Resp Cell Mol Biol.

[B20] Savill JS, Wyllie AH, Henson JE, Walport MJ, Henson PM, Haslett C (1989). Macrophage phagocytosis of aging neutrophils in inflammation. Programmed cell death in the neutrophil leads to its recognition by macrophages. J Clin Invest.

[B21] Dockrell DH, Lee M, Lynch DH, Read RC (2001). Immune-mediated phagocytosis and killing of Streptococcus pneumoniae are associated with direct and bystander macrophage apoptosis. J Infect Dis.

[B22] Bingle CD, Craig RW, Swales BM, Singleton V, Zhou P, Whyte MKB (2000). Exon skipping in Mcl-1 results in a Bcl-2 homology domain 3 (BH3)-only gene product that promotes cell death. J Biol Chem.

[B23] http://frodo.wi.mit.edu/cgi-bin/primer3/primer3_www.cgi.

[B24] Gray PW, Flaggs G, Leong SR, Gumina RJ, Weiss J, Ooi CE, Elsbach P (1989). Cloning of the cDNA of a human neutrophil bactericidal protein. Structural and functional correlations. J Biol Chem.

[B25] Bartlett JA, Hicks BJ, Schlomann JM, Nauseef WM, Whitney PL, McCray PB (2006). PLUNC Is a Secreted Product of Neutrophil Granules. Proc Am Thorac Soc.

[B26] Moulding DA, Akgul C, Derouet M, White MR, Edwards SW (2001). BCL-2 family expression in human neutrophils during delayed and accelerated apoptosis. J Leukoc Biol.

[B27] Theilgaard-Monch K, Jacobsen LC, Borup R, Rasmussen T, Bjerregaard MD, Nielsen FC, Cowland JB, Borregaard N (2005). The transcriptional program of terminal granulocytic differentiation. Blood.

[B28] Bingle L, Cross SS, High AS, Wallace WA, Rassl D, Yuan G, Hellstrom I, Campos MA, Bingle CD (2006). WFDC2 (HE4): A potential role in the innate immunity of the oral cavity and respiratory tract and the development of adenocarcinomas of the lung. Respiratory Research.

[B29] Lindahl M, Stahlbom B, Tagesson C (2001). Identification of a new potential airway irritation marker, palate lung nasal epithelial clone protein, in human nasal lavage fluid with two-dimensional electrophoresis and matrix-assisted laser desorption/ionization-time of flight. Electrophoresis.

[B30] Ghafouri B, Stahlbom B, Tagesson C, Lindahl M (2002). Newly identified proteins in human nasal lavage fluid from non-smokers and smokers using two-dimensional gel electrophoresis and peptide mass fingerprinting. Proteomics.

[B31] Cole AM, Liao H, Stuchlik O, Tilan J, Pohl J, Ganz T (2002). Cationic polypeptides are required for antibacterial activity of human airway fluid. J Immunol.

[B32] Ghafouri B, Irander K, Lindbom J, Tagesson C, Lindahl M (2006). Comparative proteomics of nasal fluid in seasonal allergic rhinitis. J Proteome Res.

[B33] Cowland JB, Borregaard N (1999). The individual regulation of granule protein mRNA levels during neutrophil maturation explains the heterogeneity of neutrophil granules. J Leukoc Biol.

[B34] Kim CH, Kim K, Jik Kim H, Kook Kim J, Lee JG, Yoon JH (2006). Expression and regulation of PLUNC in human nasal epithelium. Acta Otolaryngol.

